# Notes and News

**Published:** 1888-03-03

**Authors:** 


### NOTES AND NEWS.

Collected and Communicated.

The next meeting of the Hospitals Association will be held on Wednesday, 14th March, when Mr. T. Ryan, secretary of St. Mary's Hospital (late of Queen Charlotte's Hospital), will read a paper on "The Origin, History, and Present State of Metropolitan Lying-in Hospitals." The chair will be taken at 8 p.m. by W. S. Playfair, Esq., M.D., LL.D., etc. The reading of the paper of Mr. Burdett-Coutts, M.P., on "Contributions by Patients in Relation to the Financia Condition of London Hospitals," has been postponed until April 25th next.

The Treasury has agreed to grant £400 for the extension of the Aberdeen Medical School.

The treasurer of Guy's Hospital has received an anonymous donation of £1,000 from "H. A. N." towards the £100,000 for which a special appeal has been made.

Miss Emma G. Pease has been obliged through ill-health to resign the secretaryship of the Ladies' Committee of the Darlington Hospital, a post which she has filled for many years with great benefit to the institution.

The Dublin Corporation has voted a grant of £100 a-year to the National Lying-in Hospital, Holles Street, a gift which has saved that institution from considerable pecuniary embarrassment.

Dr. Isaac Bayley Balfour, Sherardian Professor at Oxford, has been appointed to the vacant Chair of Botany in Edinburgh University as successor to the late Professor Dickson. Professor Balfour is really going home in returning to Edinburgh and filling the position of botanical professor there. His father held the same post for many years, and was the new professor's instructor in the science they have both chosen to follow.

While the value of electricity in the treatment of various diseases has long been known, and has aroused public interest, there has been but a scanty supply of suitable apparatus to be met with in our hospitals or elsewhere. This has given a grand opportunity for quacks of all sorts, and electric belts, electric hair-brushes—every article which could have one or two pieces of metal fastened to it and be dubbed electric—has been used to wheedle money out of the pockets of the ignorant. This has inflicted a double wrong; swindling the deluded purchasers, and bringing discredit on the indubitable medical value of electricity. We therefore welcome the formation of an Institute of Medical Electricity, founded by electricians of repute and approved by eminent medical men, who will have the right to visit the Institute at all times and see that the work is conducted on the lines stated in the prospectus, which make a point of all the treatment being in accordance with medical and electric science—not guesswork. The Institute proposes not only to treat patients on the premises, but to train operators and to lend apparatus to medical men in the country and elsewhere who cannot conveniently send their patients to hospitals. The Institute supplies a want, and ought to be successful.

By invitation of the architects, Messrs. Keith D. Young and Henry Hall, the new buildings of the Great Northern Central Hospital were viewed by a number of gentlemen interested in the institution on Saturday the 25th ult.

The authorities of the Royal Lunatic Asylum at Morningside, Edinburgh, have purchased Craig House, an ancient manorial residence adjoining the grounds of the original institution. Here they intend to erect a model asylum, provided with every modern improvement, at an estimated cost of £60,000.

At the annual meeting of the Southport Infirmary several clergymen complained that the date of the Hospital Sunday collection had been fixed without notice to them. Mr. J. H. Ellis suggested that the date should be reconsidered, as the hospital could not afford to lose "such excellent beggars" as the clergy by any disregard of their convenience.

Alderman Pink, of Portsmouth, has protested against the action of the Corporation in making a donation of £50 to the hospital, and proposing to do the same every year. He considers that such a gift cannot legally be made out of the public funds; but the Mayor and Council are determined that, whether legal or not, such a subsidy is right, and Mr. Pink had only two followers in voting against it. Still, it is a pity that the vote in favour of the donation was not unanimous.

There was a crowded attendance on Wednesday last at the Whitehall Rooms of the Hôtel Métropole, Charing Cross, when the sixty-seventh Annual Court of the Seamen's Hospital Society was held. The Right Hon. Lord Charles Beresford, M.P., C.B., R.N., presided, and was supported by a distinguished gathering of ladies and gentlemen. In addressing the meeting, Lord Charles Beresford, who was very warmly cheered, expressed the opinion that the thoroughly English character with which the Seamen's Hospital wa imbued was shown by the fact that its motto was "Free to the whole maritime world." Any seaman, if he was sick, had only to show his certificate to be instantly admitted within the walls of the institution, and the characteristics of the people of these isles—kindness of heart and sympathy with those in distress—were amply demonstrated in this single fact. He reminded his hearers that the hospital was first started when the Admiralty gave the *Grampus*, an old brig, and then gave the *Dreadnought*. She became too small, and the sanitary arrangements and other matters on board a larger vessel which was given, led to the Government giving a hospital at Greenwich, which was found most excellent for the purposes for which it was provided. The society was started in 1821, and since then no fewer than 277,143 seamen had been received in it, representing 42 nationalities. There was an average of 190 beds occupied daily, and last year 2,382 seamen were relieved. His Lordship drew attention to the practical objects of the meeting, and warmly urged the great merits of the society to public support, pointing out, in a characteristic and eloquent address, the sterling character and value of the men who, he said, carried out the great maritime and commercial interests of this country. The great number of 614,000 vessels entered and left English ports in one year, and out of the large number of men which these contained, 2,568 lost their lives. Their gallant deeds in appalling scenes only rarely reached the ears of the general public, while thousands really occurred. When Jack fell sick, he found a ready welcome and kind friends at Greenwich, and he entreated his hearers to subscribe and to gain subscriptions to what he was personally certain was an admirable charity. Admiral Sir G. P. Hornby, G.C.M., Mr. T. M. Waller (Consul-General for the United States), Admiral Sir Claude Buckle, and General Erskine also spoke of the merits and claims of the hospital.

THE following vacancies are announced this week, the amount of salary in each case being given after the name of the institution :—

**Resident Medical Officers.**

Cancer Hospital, Brompton. Registrar.—£50 per annum, with board.
Coton Hill Lunatic Hospital. Assistant Medical Officer.—£100 per annum, with board
Dewsbury and District General Infirmary. House Surgeon.—£80, with board.
French Hospital, Leicester Square.—£60 per annum, with board.
Leicester Infirmary and Fever House. Assistant House Surgeon. £50, with board.
Lincolnshire County Asylum, Bracebridge, near Lincoln. Assistant Medical Officer—£150 per annum, with board, &c.
Metropolitan Hospital, Kingsland Road. Junior House Surgeon. —£40, with board.
Roxburgh District Asylum, Melrose. Assistant Medical Officer. £80 per annum, with board.

**Non-resident Medical Officers.**

Bristol Dispensary. Surgeon.
Central London Ophthalmic Hospital, Gray's Inn Road.—Two Assistant Surgeons.
Mountmellick Union, Coolrain Dispensary Medical Officer — £115 per annum, and fees.
Oughterard Union. Medical Officer, Oughterard Dispensary.—£112 per annum, and fees.
St. George's and St. James's Dispensary. Physician.

**Nurses.**

Consumption Hospital Ventnor. Superintendent of Nurses.—£30.
Rural Hospital, Tewkesbury. One.—£20
Bradford Eye and Ear Hospital. One, Trained.—£25.
Institution of Trained Nurses, Leicester. Several, Medical, Surgical, and Monthly.
Nursing Institution, Burton-on-Trent. One, Medical and Surgical.—£25.
Children's Hospital, Cardington, near Bedford. One.
Peterborough Infirmary. One.
Cottage Hospital, Batley, Yorkshire. One —£20.
Swansea and South Wales Nursing Institution. Several, Medical and Surgical.
Nursing Institution, Cheltenham. Several, Medical, Surgical, and Monthly.

**Probationer.**

Peterborough Infirmary. One.

---

AT the close of 1886 the accounts of Wrexham Infirmary showed a deficit of £308 2*s.*, which has now been cleared off almost entirely, only £41 6*s.* 8*d.* remaining. There has been a falling-off in the number of out-patients attending the infirmary, but this is probably due to the improvement of the sanitary conditions under which the poor live—a most satisfactory reason.

---

THE secretary of the Hull Royal Infirmary has been ex plaining to the subscribers that old linen, which cannot be purchased, is in continual demand there. We wish this fact could be borne in on the minds of all housekeepers, who could thus often make a useful present to the hospitals without any cost to themselves.

---

THE death of Dr. Anna Kingsford, which took place on Tuesday, the 21st ult., is a great loss to our small body of medical women. She was one of the best known among them to the public at large, and it may be surmised that her little book on the toilet, which proved that she did not frown on those accessories to beauty in which, whether forbidden or no, women will indulge, did more to reconcile her sex to "lady doctors" than the most brilliant scientific attainments could have done. Mrs. Kingsford began her medical studies in 1874, and took the degree of M.D. at Paris in 1880, her graduation thesis being on "Vegetables as Food for Man." She was a strict vegetarian herself, and attributed her being so long able to withstand the consumption to which she has at last succumbed to her strict adherence to vegetable diet. She was in every sense a humanitarian—a keen anti-vivisectionist, and a practical protestant against killing animals for the sake of their fur. Indeed, with all her intellectual qualities and independence of judgment, Dr. Anna Kingsford was a thoroughly womanly woman.

THE Executive Committee of the Victoria Jubilee Hospital, Bournemouth, in their first annual report, recognise with great thankfulness the handsome and liberal support that has been accorded to them by the Bournemouth public. It is to be hoped that those to whom these grateful words are addressed will continue to deserve them year by year.

---

MR. FORSTER GREEN offered to bear the cost of building a wing to the Throne Hospital, Belfast, on condition that a sum of £15,000 could be raised as an endowment. As the best efforts of the hospital authorities have gained only about half the necessary sum, Mr. Green has withdrawn his offer, but purposes applying the sum he intended for the building to the relief of consumptive patients, through private channels.

---

THE Bourton-on-the-Water Cottage Hospital maintains a course of steady prosperity. On the 31st December, 1887, a balance of nearly £44 stood to the credit of the hospital in the bank at Stow. The number of patients treated during the year was thirty-eight, rather under the average of past years; for which decrease the warm and dry summer we enjoyed may probably account. Dr. W. C. Coles is still, as he has been since its erection in 1879, the honorary secretary to the hospital.

---

AN arrangement has been entered into by the Liverpool dispensaries with the School Board of the town, by which parents can, without difficulty or expense, obtain medical certificates as to the condition of their children when they are, on account of illness, unable to attend school. This is a good idea, by which the School Board will profit, as there can be no longer any loophole for defaulting parents who keep their children at home for any reason or none, and protest, when they plead illness, that they could not afford the certificate they ought to produce as a voucher for the truth of their statement.

---

THE quarterly General Court of the Middlesex Hospital was held on the 22nd ult., Mr. R. Ruthven Pym presiding. The annual report and accounts were adopted, and the annual officers re-elected. Lord Sandhurst drew attention to the finances, which show an income of £15,213 to an expenditure of £26,758, leaving a deficiency of £11,500 to be made up out of capital. One of the special recommendations of this hospital to public benevolence is its cancer ward, where 34 patients, received without letters of recommendation or any other claim than their need of help, are cared for till their sufferings are ended by skill or death. Mr. F. Clare Melhado was appointed secretary-superintendent to the Middlesex Hospital on the 21st ult.

---

THE annual meeting of the East London Nursing Society was held at the Mansion House on Tuesday, the 28th ult. The Lord Mayor presided during the first part of the proceedings, but being compelled by other business to leave before the meeting terminated, he left behind him, as a token of his interest in the work of the Society, a cheque for ten guineas. After his departure the Rev. Harry Jones took the chair. Besides Mr. Jones, there were present: The Rev. Mr. Hoskins (rector of Stepney), the Rev. W. P. Jay (vicar of Christchurch), Sir Dyce Duckworth, Dr. Steell, and Dr. Owen Lankester, all of whom bore eloquent testimony to the good work done by the Society in providing trained nurses to tend the sick poor of the East End in their own homes. During last year the annual subscriptions increased from £591 6*s.* 6*d.* to £738 8*s.* 6*d.*, and by the kindness of one donor the committee were enabled to provide a nurse for the parish of St. Matthew's, Stepney. The cost of establishing a nurse is not less than £60 for the first year, and about £54 per annum afterwards. We purpose giving next week an account of the work done by this Society, as observed by our Commissioner.

